# The Editor's Letter-Box

**Published:** 1920-11-20

**Authors:** 


*To the Editor of* THE HOSPITAL.

*To the Editor of* THE HOSPITAL.

SIR,—The pendulum has swung far—a few years ago almoners were generally regarded as curiously hard-hearted officials, this idea being based mainly on the fact that, instead of giving free treatment, free instruments, and free convalescence, unconditionally to hospital patients, their custom was to ask the patients to contribute according to his means. The underlying principle of their work, and consequently their practice, remains the same, but the cry now is that the almoners are too soft-hearted, and THE HOSPITAL of October 30 states, with surprise and disapproval, that almoners are not "in love" with the system of patients' contributions.

Would it not be fairer to say that they are not unduly inflated, even if the principle they have preached so long is now proclaimed from the house-tops?

As THE HOSPITAL so wisely says, almoners realise that it is more blessed to give than to receive, but, just because they realise it, they have never tried to reserve blessedness to themselves, but have shared it ungrudgingly with the patients!

In well-staffed hospitals the almoners are working valiantly to collect patients' donations, with most excellent results—in other hospitals, where the almoners' department is understaffed, it is a physical impossibility for them to do this work as it should be done, in addition to their other labours.

It is surely a truism, however, to say that the almoner is the ideal person for the job. Her whole training inculcates balance and sound judgment. She has the welfare of the patient and the welfare of the hospital equally at heart—the hospital must have all the support possible—the patient must give, but not to the detriment of his health. As THE HOSPITAL says, a difficult time of unemployment is probably at hand, so that the work of collecting payments will need wisdom and knowledge. The almoners realise the importance of the task, and are prepared to do their utmost to maintain the voluntary spirit in the hospital and to inculcate the value of self-help amongst the patients.—Yours faithfully,

"A TRAINED ALMONER."

